# Physical Exercise as a Non-Pharmacological Intervention for Attenuating Obesity-Related Complications in Children and Adolescents

**DOI:** 10.3390/ijerph19095046

**Published:** 2022-04-21

**Authors:** Valeria Calcaterra, Gianvincenzo Zuccotti

**Affiliations:** 1 Department of Pediatrics, “Vittore Buzzi” Children’s Hospital, 20157 Milano, Italy; gianvincenzo.zuccotti@unimi.it; 2 Department of Internal Medicine and Therapeutics, University of Pavia, 27100 Pavia, Italy; 3 Department of Biomedical and Clinical Science “L. Sacco”, University of Milano, 20157 Milano, Italy

Obesity is the most prevalent nutrition-related disorder among the pediatric population. The World Health Organization (WHO) estimated that about 41 million children aged < 5 years and 340 million subjects aged 5–19 years are affected by overweight or obesity [1]. Physical activity (PA) and the promotion of an active lifestyle play fundamental roles among children and adolescents in reducing the negative effects of weight gain [2]. Although obesity is a multifactorial disease, physical inactivity and a sedentary lifestyle are the key players in the growing rates of pediatric obesity [3]. 

Childhood obesity is a multisystem condition that has potentially harmful consequences and various comorbidities, thereby contributing to premature mortality in adulthood [4]. In addition to cardio-metabolic obesity-related disorders and psychological problems, recent evidence suggests that obesity affects also immune system function, leading to systemic low-grade chronic inflammation (SLGCI) [4].

Adipose tissue (AT) immunomodulation is a crucial pathogenetic factor of all obesity comorbidities. Adipocyte hypertrophia and/or hyperplasia can promote local hypoxia, mechanical stress, and death of the adipocytes inducing the secretion of pro-inflammatory molecules and chemokines by adipocytes, endothelial cells, and resident macrophages, thus initiating an inflammatory process and transition of this local inflammation to SLGCI [5]. SLCGI is associated with human ageing. Although ageing is a process usually described in elderly subjects, this concept should be also considered in pediatrics when considering subjects with high cumulative biological risk, such as children with obesity [5]. The initiating events of inflammation start early in childhood and modulate interconnected biochemical molecular pathways including stress adaptation, epigenetics, inflammation, macromolecular damage, metabolism, proteostasis, and stem cell and tissue regeneration, leading to related morbidity and mortality risk factors [6].

Sedentary children and adolescents are at higher risk for cardio-metabolic diseases than subjects who engage in regular physical exercise (PE) [7]. Less than 20% of adolescents in the world are sufficiently physically active [7]. As recommended by WHO in 2020, promoting an active lifestyle and appropriate levels of physical activity is mandatory to reduce the risk of preventable adverse health events [7]. 

PE is a non-pharmacological intervention that can delay obesity-related comorbidities, improving cardiovascular fitness and attenuating SLGCI.

Several beneficial PE effects have been described [8,9,10,11,12]. Firstly, PE induces an increase in exercise tolerance supported by an increase in cardiac and skeletal muscle strength, as well as improvements in maximal oxygen consumption [8,9]. Secondly, PE impacts the metabolic profile. In particular, PE influences insulin sensitivity in AT, skeletal muscle, and the endothelium, leading to decreased insulin resistance risk; PE also reduces body weight and blood pressure (increasing vascular arteriolar density and reducing systemic vascular resistance) and induces an increase in the HDL cholesterol particle size, as well as a decrease in the VLDL particle size [8,10]. PE also improves the immunological response with a delayed immunosenescence onset and reduced SLGCI [9]. Indeed, a low PA is associated with a reduced release of cytokines and myokines produced by muscular contractions, thereby attenuating systemic inflammation [10,11]. Additionally, microbiome dysbiosis has been reported in patients with obesity to promote negative metabolic effects and inflammation. PE was also reported to be a significant modifier in preventing and restoring gut dysbiosis [12]. Finally, considering the relationship between PA and mental health, PA’s benefits for psychological problems must also be considered. 

PE may represent a tool to mitigate the global problems of overweight and obesity, as well as related comorbidities. Regular PE must be considered to be a natural part of a healthy lifestyle starting from childhood. Public health campaigns that promote the role of PE are crucial to support the care and wellbeing of pediatric population. Promoting PA is also needed to limit the negative effects of obesity on health economics. To consider the potential effects of PA and PE and the pathogenic mechanisms by which PA impacts the resolution of comorbidities must be explored to propose and adopt a new preventive perspective in children and adolescents. The role of PE as a non-pharmacological intervention for attenuating obesity-related complications should be considered and supported by healthcare providers, teachers, parents and guardians to ameliorate the future burden of pediatric obesity comorbidities.
